# Symmetries and integrable systems

**DOI:** 10.1016/j.fmre.2023.11.008

**Published:** 2023-12-04

**Authors:** Sen-Yue Lou, Bao-Feng Feng

**Affiliations:** a School of Physical Science and Technology, Ningbo University, Ningbo 315211, China; b School of Mathematical and Statistical Sciences, The University of Texas Rio Grande Valley, Edinburg, TX 78539, United States

**Keywords:** Symmetries, Integrable systems, Recursion operators, Formal series symmetries, Nonlocal symmetries, Darboux and Bäcklund transformation, Exact solutions

## Abstract

Symmetry plays key roles in modern physics especially in the study of integrable systems because of the existence of infinitely many local and nonlocal generalized symmetries. In addition to the fundamental role to find exact group invariant solutions via Lie point symmetries, some important new developments on symmetries and conservation laws are reviewed. The recursion operator method is important to find infinitely many local and nonlocal symmetries of (1 + 1)-dimensional integrable systems. In this paper, it is pointed out that a recursion operator may be obtained from one key symmetry, say, a residual symmetry. For (2 + 1)-dimensional integrable systems, the master-symmetry approach and the formal series symmetry method are reviewed. For the discrete systems, the symmetry related discrete KP hierarchy and the BKP hierarchy are also discussed. One believes that all the solutions of integrable models may be obtained by means of symmetry approach because the Darboux transformations and algebro-geometric solutions can be obtained from the localization of nonlocal symmetries and the symmetry constraint approach. The conservation laws are used to find higher dimensional integrable system from lower dimensional ones via a deformation algorithm. The ren variable, an extension of the Grassmann variable, are introduced to find novel aspect on integrable theory. The super-integrable theory and super-symmetric integrable theory are extended to ren integrable and ren-symmetric integrable theories.

## 1 Introduction

Symmetry plays a key role in almost all the fields of physics especially in integrable systems because of the existence of infinitely many local and nonlocal generalized symmetries [1], [2], [3], [4], [5], [6]. Symmetries are usually used to find symmetry invariant solutions, similarity reductions and even all solutions of nonlinear systems [7]. Noether's theorem points out that every continuous symmetry is closely related to a conservation law. In this short review paper, we point out some other aspects on the symmetries of integrable systems.

In Section 2, we review the fundamental role of the Lie point symmetries of a nonlinear system to find its group invariant solutions. To find generalized symmetries of (1 + 1)-dimensional integrable systems, the most powerful method is to find the so-called recursion operators. Section 3 includes a simple method to find recursion operators and then the generalized local and nonlocal symmetries of (1 + 1)-dimensional integrable systems. Using the localization of nonlocal symmetries for integrable systems, one can obtain Darboux transformations and many interaction solutions among solitons and some other types of nonlinear waves such as the Painlevé waves and cnoidal periodic waves. Applying the symmetry constraint method, the algebro-geometric solutions of integrable systems can be obtained by solving finite dimensional integrable systems. In Section 4, the master-symmetry approach and the formal series symmetry method are applied to find general symmetries for (2 + 1)-dimensional integrable systems. Section 5 is devoted to discuss the symmetries of discrete integrable systems. In Section 6, the conservation laws are used to find higher dimensional integrable systems thanks to a deformation algorithm. Using the deformation algorithm to any (1 + 1)-dimensional integrable systems, one can obtain some higher dimensional integrable models because of the existence of infinitely many symmetries and then conservation laws. In Section 7, the nonlocal symmetries related to the spectral functions of the Lax integrable systems are used to find new integrable systems related to the different nonlinear fields. The spectral functions usually are considered as bosonic. In this case, the integrable interacting models are called the integrable systems with consistent sources [8], [9], [10]. For the KdV equation, the integrable model with sources can be considered as the interacting model among long wave and short waves. In fact, the spectral functions may be fermionic and even any other non-commute fields, say, the ren fields which will be introduced in this paper. Whence the fermionic spectral functions are considered, the integrable model with sources are just the super- or Kuper-integrable systems [11]. If the spectral functions are taken as ren functions, then the integrable model with self-consistent sources will be defined as ren-integrable models which are the generalization of the super-integrable models. In Section 8, it is pointed out that the significant supersymmetric integrable systems can be extended to the so-called ren-symmetric integrable systems. The last section of the paper includes a short summary and some discussions.

## 2 Lie point symmetries of nonlinear partial differential equations

To find some explicit exact solutions of nonlinear partial differential equations (PDEs), the classical and nonclassical symmetry approach is one of the most popular and universal method no matter the PDEs are integrable or not. In fact, one can use some existing software to find exact solutions related to Lie point symmetries [12]. Here, we just take a simple example, the Korteweg-de Vries (KdV) equation, to explain the fundamental procedure to find Lie point symmetries and the related finite transformations, group invariant solutions and symmetry reductions of nonlinear systems without using any packages.

For the KdV equation:

(1) $$u_{t}+u_{xxx}-6uu_{x}=0$$

a symmetry, σ, is defined as a solution of its linearized equation:

(2) $$\sigma_{t}+\sigma_{xxx}-6\sigma u_{x}-6u\sigma_{x}=0$$

that means Eq. 1 is form invariant under the transformation u→u+εσ with infinitesimal ε. A Lie point symmetry is defined as if a symmetry possesses the form:

(3) $$\sigma =U\left(x,t,u\right)-X\left(x,t,u\right)u_{x}-T\left(x,t,u\right)u_{t}\;$$

After substituting Eq. 3 into Eq. 2 and cancelling $u_{t}$ by means of Eq. 1, it is not difficult to find the final Lie point symmetry of the KdV equation has the form:

$$\sigma =2cu+g+\left(cx+6gt+x_{0}\right)u_{x}+\left(3ct+t_{0}\right)u_{t}$$

$$=c\left(2u+xu_{x}+3tu_{t}\right)+g\left(1+6tu_{x}\right)+x_{0}u_{x}+t_{0}u_{t}$$

(4) $$=c\tau_{1}+g\tau_{0}+x_{0}K_{0}+t_{0}K_{1}$$

with arbitrary constants $c,\;g,\;x_{0}$ and $t_{0},$ which are related to the scaling invariance, the Gallian invariance, the space translation invariance and the time translation invariance, respectively.

The finite transformation of Eq. 4 can be simply obtained by solving the initial valued problem:

$$\frac{dx_{1}}{d\varepsilon }=cx_{1}+6gt_{1}+x_{0},\;\frac{dt_{1}}{d\varepsilon }=3ct_{1}+t_{0},\;\frac{du_{1}}{d\varepsilon }=-2cu_{1}-g$$

(5) $$u_{1}\left(0\right)=u,\;x_{1}\left(0\right)=x,\;t_{1}\left(0\right)=t\;$$

The general solution of Eq. 5 read:

(6) $$u_{1}=e^{-2c\varepsilon }u+g_{0},\;x_{1}=e^{c\varepsilon }x-6e^{3c\varepsilon }g_{0}t+X_{0},\;t_{1}=e^{3c\varepsilon }t+T_{0}$$

In Eq. 6, the constants $\left\{g_{0},X_{0},T_{0}\right\}$ are given by:

(7) $$T_{0}=\frac{a^{3}-1}{3c}t_{0},\;X_{0}=\frac{a-1}{c}x_{0}+\frac{a^{3}-3a+2}{c^{2}}gt_{0},\;g_{0}=\frac{1-a^{2}}{2ca^{2}}g,\;a=e^{c\varepsilon }\;$$

The equivalent expression of Eq. 6 is that if u=u(x,t) is a solution of the KdV Eq. 1, then:

(8) $$u_{1}=e^{-2c\varepsilon }u\left(e^{-c\varepsilon }\left(x+6g_{0}t-X_{0}-6g_{0}T_{0}\right),\;\;e^{-3c\varepsilon }\left(t-T_{0}\right)\right)+g_{0}$$

is also a solution.

The invariant solution of the KdV Eq. 1 related to the symmetry Eq. 4 can be obtained by solving Eq. 4 with σ=0. The result can be separated to two cases.

Case 1. c≠0. In this case, the solution of σ=0 reads:

(9) $$u=-\frac{g}{2c}+\frac{U\left(X\right)}{{\left(3ct+t_{0}\right)}^{\frac{2}{3}}},\;X=\frac{c^{2}x+cx_{0}-2gt_{0}}{c^{2}{\left(3ct+t_{0}\right)}^{\frac{1}{3}}}-\frac{g}{c^{2}}{\left(3ct+t_{0}\right)}^{\frac{2}{3}}$$

where U(X) satisfies the following third order ordinary differential equation:

(10) $$U_{XXX}=6UU_{X}+cXU_{X}+2cU\;$$

which is equivalent to the Painlevé II equation [13,14].

Case 2. c=0. In this case, we have:

(11) $$u=U\left(Y\right)-\frac{gt}{t_{0}}-\frac{x_{0}}{6t_{0}},\;Y=x-\frac{3gt^{2}}{t_{0}}-\frac{x_{0}t}{t_{0}}\;$$

where U satisfies:

(12) $$U_{YY}=3U^{2}+\frac{gY}{t_{0}}+c_{0}\;$$

which is equivalent to the Painlevé I equation for g≠0. When g=0, Eq. 12 can be solved by means of the Weierstrass elliptic functions.

## 3 Recursion operators and generalized local and nonlocal symmetries

In addition to the Lie point symmetries, there are infinitely many generalized symmetries for integrable systems. In (1 + 1)-dimensional cases, to find infinitely many generalized symmetries, one can apply strong symmetries and/or recursion operators.

Definition 1 For a known symmetry $\sigma_{0}$ of a PDE $u_{t}=F\left(u\right)$, if $\Phi \sigma_{0}$ is also a symmetry, then the operator Φ is defined as a strong symmetry of the PDE.

Definition 2 If the operator Φ is not only a strong symmetry of $u_{t}=F\left(u\right),$ but also a strong symmetry of the PDE $u_{t}=\Phi F\left(u\right)$, then we call Φ is a hereditary operator or a recursion operator [1,15].

It is not difficult to verify that the operator:

(13) $$\Phi =\partial_{x}^{2}-4u-2u_{x}\partial_{x}^{-1}$$

is just the recursion operator of the KdV Eq. 1 and:

(14) $$K_{n}=\Phi^{n}K_{0},\;\;\tau_{n}=\Phi^{n}\tau_{0},\;n=0,\;1,\;2,\ldots ,\;\infty \;$$

where $K_{0},\;K_{1},\;\tau_{0},\;\tau_{1}\;$ are just the known Lie point symmetries defined in Eq. 4. $K_{n}\;\text{and}\;\tau_{n}$ for n≥2 are local generalized symmetries, say, for n=2:

(15) $$\;K_{2}={\left(10uu_{xx}+5u_{x}^{2}-10u^{3}-u_{xxxx}\right)}_{x}$$

The generalized symmetries may be local and nonlocal. To find generalized nonlocal symmetries, there are various methods including the factorization of inverse recursion operators [16], the infinitesimal Darboux transformations [17], the infinitesimal Bäcklund transformations [18] and the residual symmetries [19]. Here, we apply the truncated Painlevé analysis [20] to derive the residual symmetry for the KdV Eq. 1.

After finishing some standard calculations for the Painlevé analysis of the KdV equation, we have the auto-Bäcklund transformation:

(16) $$u=\frac{2f_{x}^{2}}{f^{2}}-\frac{2f_{xx}}{f}+u_{2}$$

where u and $u_{2}$ are all the solutions of the KdV Eq. 1 while the function f is a solution of the singular manifold equation, i.e., the Schwarz KdV equation:

(17) $$f_{t}=-f_{xxx}+\lambda f_{x}+\frac{3f_{xx}^{2}}{2f_{x}}$$

and $u_{2}$ is related f by:

(18) $$u_{2}=-\frac{f_{xx}^{2}}{4f_{x}^{2}}+\frac{f_{xxx}}{f_{x}}+\frac{\lambda }{6}$$

It is clear that Eq. 18 is a nonauto-Bäcklund transformation between the KdV Eq. 1 and the Schwarz KdV Eq. 17. It is also interesting that the residual, $-2f_{xx}$, given in Eq. 16 with respect to the singular manifold *f* is just a nonlocal symmetry of the KdV Eq. 1 with $u=u_{2}\;$ [19,20]. Thus, applying the inverse of the recursion operator Eq. 13:

(19) $$\Phi^{-1}=\partial_{x}f_{x}\partial_{x}^{-1}f_{x}^{-1}\partial_{x}^{-1}f_{x}^{-1}\partial_{x}^{-1}f_{x}$$

on the residual symmetry, one can find a set of nonlocal symmetries:

(20) $$K_{-n-1}^{\left(0\right)}=\Phi_{x}^{-n}K_{-1}^{\left(0\right)},\;\;K_{-1}^{\left(0\right)}=f_{xx},\;n=0,\;1,\;2,\ldots ,\;\infty \;$$

In fact, the residual symmetry is a kernel of the recursion operator Φ. From the expression Eq. 19, we know that there are three kernels of Φ. One can check that the other two kernels:

(21) $$\;K_{-1}^{\left(1\right)}=\partial_{x}f_{x}\partial_{x}^{-1}f_{x}^{-1}=f_{xx}\partial_{x}^{-1}f_{x}^{-1}+1$$

and

(22) $$K_{-1}^{\left(2\right)}=\partial_{x}f_{x}\partial_{x}^{-1}f_{x}^{-1}\partial_{x}^{-1}f_{x}^{-1}=\frac{1}{2}f_{xx}{\left(\partial_{x}^{-1}f_{x}^{-1}\right)}^{2}+\partial_{x}^{-1}f_{x}^{-1}$$

are all the nonlocal symmetries of the KdV Eq. 1. Therefore, we have three sets of infinitely many nonlocal symmetries:

(23) $$K_{-n-1}^{\left(i\right)}=\Phi_{x}^{-n}K_{-1}^{\left(i\right)},\;n=0,\;1,\;2,\ldots ,\;\infty ,\;i=0,\;1,\;2$$

which are equivalent to those given in Ref. [16].

The nonlocal symmetries, $f_{ixx},\;i=1,\;2,\;\ldots ,\;n,$ can be localized with help of the localization approach of nonlocal symmetries by extending the KdV solution space {*x, t, u*} Eq. 1 to the enlarged space {*x, t, u,* $f_{i}$*,* $g_{i}$*,* $h_{i}$}, where, $f_{i},\;i=1,\;2,\;\ldots ,\;n,$ are related to *u* by:

(24) $$u=-\frac{f_{ixx}^{2}}{4f_{ix}^{2}}+\frac{f_{ixxx}}{f_{ix}}+\frac{\lambda_{i}}{6},\;g_{i}=f_{ix},\;h_{i}=g_{ix}$$

Using the *n* copies of the nonlocal symmetries $f_{ixx}$ in its vector form:

(25) $$V=2\sum_{i=1}^{n}c_{i}f_{ixx}\partial_{u}$$

one can derive the *n*-fold Darboux and/or Bäcklund transformations by using localization approach [19,20]. The localized form of (25) reads [20]:

$$V=2\sum_{i=1}^{n}c_{i}h_{i}\partial_{u}-\sum_{i=1}^{n}\left[c_{i}f_{i}^{2}+\sum_{j\neq i}^{n}\frac{c_{j}}{4}\frac{{\left(g_{i}h_{j}-h_{i}g_{j}\right)}^{2}}{g_{i}g_{j}{\left(\lambda_{i}-\lambda_{j}\right)}^{2}}\right]\partial_{f_{i}}$$

$$\;-\sum_{i=1}^{n}\left[2c_{i}g_{i}f_{i}+\sum_{j\neq i}^{n}c_{j}\frac{g_{i}h_{j}-h_{i}g_{j}}{\lambda_{i}-\lambda_{j}}\right]\partial_{g_{i}}$$

(26) $$\;-\sum_{i=1}^{n}\left\{2c_{i}\left(h_{i}f_{i}+g_{i}^{2}\right)+\sum_{j\neq i}^{n}\frac{c_{j}}{2}\left[4g_{i}g_{j}+\frac{g_{i}^{2}h_{j}^{2}-h_{i}^{2}g_{j}^{2}}{\left(\lambda_{i}-\lambda_{j}\right)g_{i}g_{j}}\right]\right\}\partial_{h_{i}}$$

Starting from the vector form of the localized symmetry Eq. 26 of the nonlocal symmetry Eq. 25, we can find its finite form, i.e., the Darboux and/or Bäcklund transformation of the KdV Eq. 1.

*Darboux transformation theorem*. If $\;\{u,\;f_{i},\;g_{i},\;h_{i}\}$ is a solution of the prolonged KdV system (1), (24) and

(27) $$f_{it}=-f_{ixxx}+\lambda_{i}f_{ix}+\frac{3f_{ixx}^{2}}{2f_{ix}}$$

so is $\left\{\bar{u}=u+2{\left(\ln\Delta \right)}_{xx},\;{\bar{f}}_{i}=-\frac{\Delta_{i}}{\Delta },{\bar{g}}_{i}={\bar{f}}_{ix},{\bar{h}}_{i}={\bar{g}}_{ix}\right\}$ with $\Delta =\det\left(M\right),M_{ji}=\epsilon c_{i}w_{ji}=\epsilon c_{i}\frac{g_{i}h_{j}-g_{j}h_{i}}{2\sqrt{g_{i}g_{j}}\left(\lambda_{j}-\lambda_{i}\right)},\;j\neq i,\;M_{ii}=\epsilon c_{i}f_{i}-1$ and $\Delta_{k}=\det\left(M_{k}\right),\;M_{k,ji}=\epsilon c_{i}w_{ji},\;i\neq j,k,\;M_{k,jk}=w_{jk},\;j\neq k,\;M_{k,ii}=\epsilon c_{i}f_{i}-1,\;i\neq k,\;M_{k,kk}=f_{k}.▪$

For integrable systems, various mathematicians believe that all the solutions can be obtained by means of Darboux transformations. Now, we have obtained the Darboux transformation of the KdV equation by means of nonlocal symmetries. That means we may obtain all solutions of the KdV equation from nonlocal symmetries. It is also known that the local and nonlocal symmetries are dual [21], which implies that all solutions of the KdV equation may also be obtained by means of its local symmetries. In some times, to find exact solution it is convenience to combine local and nonlocal symmetries. For instance, if we combine the local symmetries, $\;K_{0},\;K_{1},\;\tau_{0},\;\tau_{1}$ and nonlocal symmetries Eq. 25, one can obtain various interaction solutions between the solitons and other KdV waves such as the cnoidal periodic wave and Painlevé I and II waves [17].

To find some more concrete solutions of the KdV equations, one may directly use the so-called symmetry constraint method. For instance, if we use the symmetry constraint condition $K_{0}=2\sum_{i=1}^{n}f_{ixx}$, i.e.:

(28) $$u=2\sum_{i=1}^{n}f_{ix}$$

then one can find the algebro-geometric solutions of the KdV Eq. 1 by solving the following finite dimensional integrable systems:

(29) $$f_{ix}=g_{i},\;g_{ix}=h_{i},h_{ix}=\frac{1}{4}h_{i}^{2}g_{i}^{-1}-2g_{i}\;\sum_{k=1}^{n}g_{k}-\frac{1}{6}g_{i}\lambda_{i}\;$$

and

(30) $$f_{it}=-2g_{i}\sum_{k=1}^{n}g_{k}+\frac{7}{12}\lambda_{i}g_{i}+\frac{5}{8}h_{i}^{2}g_{i}^{-1}$$

$$g_{it}=-\frac{1}{2}\sum_{k=1}^{n}\left(9h_{i}g_{k}+4g_{i}h_{k}\right)+\frac{3}{8}\lambda_{i}h_{i}-\frac{5}{16}h_{i}^{3}g_{i}^{-2}$$

$$\begin{array}{ccc} h_{it} & = & 13g_{i}{\left(\sum_{k=1}^{n}g_{k}\right)}^{2}+\sum_{k=1}^{n}\left(\frac{3}{4}\frac{g_{k}h_{i}^{2}}{g_{i}}-\frac{h_{k}^{2}}{2g_{k}}+\frac{\lambda_{k}g_{k}}{3}-\frac{13h_{i}h_{k}}{2}\right)\\ & & -\frac{\lambda_{i}^{2}g_{i}}{16}+\frac{\lambda_{i}h_{i}^{2}}{4g_{i}}+\frac{25h_{i}^{4}}{64g_{i}^{3}} \end{array}$$

The finite dimensional integrable systems Eqs. 29 and 30 are equivalent to those obtained from the nonlinearization approach [22], [23], [24]. Thus, the algebro-geometric solution Eq. 28 with Eqs. 29-30 are same as known ones as explicitly shown in [22,23] for the KP equation by eliminating the *y* variable. Because of the duality of the generalized local and nonlocal symmetries [21], the algebro-geometric solutions (28) are also same as those obtained by the local symmetry constraint, $K_{n}=\Phi^{n}K_{0}=0,$ where $K_{n}$ is defined in Eq. 14.

To end this section, we derive the recursion operator Eq. 13 from the single nonlocal symmetry $f_{ixx}$ given by Eq. 24.

Substituting $f_{ixx}=\sigma_{i}\;$ into Eq. 24, we have:

(31) $$u\sigma_{i}=\frac{1}{6}\;\sigma_{i}\lambda_{i}-\frac{1}{4}\frac{\sigma_{i}^{3}}{f_{ix}^{2}}+\frac{1}{2}\frac{\sigma_{i}\sigma_{ix}}{f_{ix}}$$

In order to cancelling the nonlinear terms of $\sigma_{i}$ in Eq. 31, multiplying Eq. 24 by $f_{ix}$ and differentiating the result with respect to *x* yields:

(32) $$u\sigma_{i}+u_{x}\partial_{x}^{-1}\sigma_{i}=\frac{1}{6}\;\sigma_{i}\lambda_{i}+\frac{1}{4}\frac{\sigma_{i}^{3}}{f_{ix}^{2}}-\frac{1}{2}\frac{\sigma_{i}\sigma_{ix}}{f_{ix}}+\frac{1}{2}\sigma_{ixx}\;$$

It is clear that Eq. 31+Eq. 32 becomes $\left(\lambda_{1i}\equiv -\frac{2}{3}\lambda_{i}\right)$:

(33) $$\left(\partial_{x}^{2}-4u-2u_{x}\partial_{x}^{-1}\right)\sigma_{i}\equiv \Phi \sigma_{i}=\lambda_{1i}\sigma_{i}$$

One can directly verify that the symmetry Eq. 2 with $\sigma =\sigma_{i}$ and the eigenvalue problem of Φ, Eq. 33 is just a Lax pair of the KdV Eq. 1. Thus, Φ derived from the residual symmetry is just the recursion operator of the KdV equation. It is interesting that one can derive recursion operator from one key nonlocal symmetry such as the residual symmetry, square-eigenfunction symmetry etc. for many (1 + 1)-dimensional integrable systems.

## 4 Master-symmetries and formal series symmetries

Expect for the breaking soliton systems, linearizable C integrable systems and higher dimensional dispersionless equations such as the heavenly equations [21,25], there is no recursion operators for higher dimensional integrable systems. To find symmetries of higher dimensional integrable systems, some other types effective methods including the master symmetry method [[21], [26]] and the formal series symmetry approach [27], [28], [29] have been developed.

### 4.1 Master symmetry method

For simplicity to illustrate the master symmetry method, we take the Kadomtsev-Petviashvili (KP) equation:

(34) $${\left(u_{t}+u_{xxx}-6uu_{x}\right)}_{x}+3\gamma^{2}u_{yy}=0$$

i.e.:

(35) $$u_{t}=-u_{xxx}+6uu_{x}-3\gamma^{2}\partial_{x}^{-1}u_{yy}\equiv K\left(u\right)$$

as a simple example. A symmetry of the KP equation, σ, is a solution of its linearized equation:

(36) $${\left(\sigma_{t}+\sigma_{xxx}-6u\sigma_{x}-6\sigma u_{x}\right)}_{x}+3\gamma^{2}\sigma_{yy}=0$$

If *Z* is not a symmetry of the nonlinear system $u_{t}=K\left(u\right)$, however, its commutator σ=[K,Z] defined by:

(37) $$K_{\left[,\right]}Z\equiv \left[K,Z\right]\equiv K^{\prime }Z-Z^{\prime }K\equiv \lim_{\epsilon \to 0}\frac{d}{d\epsilon }\left[K\left(u+\epsilon Z\right)-Z\left(u+\epsilon K\right)\right]$$

is a symmetry of $u_{t}=K\left(u\right)$, then we call *Z* a first order master symmetry. If the *k* times commutator of *Z* with respect to *K,* $K_{\left[,\right]}^{k}Z$, is a symmetry, then Z is the *kth* order master symmetry of the nonlinear system $u_{t}=K\left(u\right).$

For the KP Eq. 34, one can find that $Z=y^{n-1}$ is just *nth* order master symmetry. The first few symmetries obtained from the master symmetries read:

(38) $$\sigma_{1}=\left[K,\frac{y^{0}}{2\cdot 3}\right]=u_{x}$$

(39) $$\sigma_{2}=\left[K,\left[K,\frac{y^{1}}{2^{2}\cdot 3^{2}\cdot \gamma^{2}}\right]\right]=K_{\left[,\right]}^{2}\frac{y}{2^{2}\cdot 3^{2}\cdot \gamma^{2}}=u_{y}$$

(40) $$\sigma_{3}=K_{\left[,\right]}^{3}\frac{y^{2}}{2^{2}\cdot 3^{3}\cdot \gamma^{2}}=u_{t}$$

(41) $$\sigma_{4}=K_{\left[,\right]}^{4}\frac{y^{3}}{2^{4}\cdot 3^{5}\cdot \gamma^{2}}=4uu_{y}+2u_{x}v_{x}-u_{xxy}-\gamma^{2}v_{yy},\;u_{y}=v_{xx}$$

### 4.2 Formal series symmetry approach

To find more generalized symmetries for (2 + 1)-dimensional integrable systems, a formal series symmetry approach is proposed in Refs. [27], [28], [29] for the nonlinear systems in the form:

(42) $$u_{xt}=K_{x}\left(u\right)$$

A symmetry of (42), σ, is determined by its linearized equation:

(43) $$\sigma_{xt}=\partial_{x}K^{\prime }\sigma \;$$

A formal series symmetry of Eq. 42, i.e., a formal solution of Eq. 43 can be written as [27], [28], [29]:

(44) $$\sigma \left(f\right)=\sum_{k=0}^{\infty }f^{\left(-k\right)}K_{\left[,\right]}^{k}g\left(y\right)\;$$

where f≡f(t) and g≡g(y) are arbitrary functions of *t* and *y* respectively, and $f^{\left(i\right)}\equiv \partial_{t}^{i}f$. It is interesting that for many integrable systems such as the KP equation [28], the Toda system [27], the B-type KP equation [29] etc., the series symmetries expressed by Eq. 44 will be truncated if the function g is fixed as a polynomial function of *y*. For the KP Eq. 34 or Eq. 35, the formal series symmetry Eq. 44 becomes [27,28]:

(45) $$\sigma_{n+1}\left(f\right)=\sum_{k=0}^{n+1}f^{\left(n+1-k\right)}K_{\left[,\right]}^{k}y^{n}\;\;$$

The first four of them read:

(46) $$\sigma_{1}\left(h\right)=hu_{x}+\frac{1}{6}\overset{˙}{h}\;$$

(47) $$\sigma_{2}\left(g\right)=gu_{y}-\frac{1}{6\gamma^{2}}\overset{˙}{g}yu_{x}-\frac{1}{36\gamma^{2}}\ddot{g}y\;$$

(48) $$\sigma_{3}\left(f\right)=fu_{t}+\frac{1}{3\gamma^{2}}\overset{˙}{f}\left(xu_{x}+2yu_{y}+2u\right)+\frac{1}{18\gamma^{2}}\ddot{f}\left(x-y^{2}u_{x}\right)-\frac{1}{108\gamma^{2}}\dddot{f}y^{2}\;$$

$$\sigma_{4}\left(m\right)=m\left(4uu_{y}-u_{xxy}+2u_{x}v_{x}-\gamma^{2}v_{yy}\right)+\frac{y\overset{˙}{m}}{12\gamma^{2}}(2\gamma^{2}xu_{y}+4\gamma^{2}\partial_{x}^{-1}u_{y}$$

(49) $$-yu_{t})-\frac{y\ddot{m}}{36\gamma^{2}}\left(xu_{x}+yu_{y}+2u\right)+\frac{y\dddot{m}}{648\gamma^{4}}\left(y^{2}u_{x}-3x\gamma^{2}\right)+\frac{m^{\left(4\right)}y^{3}}{3888\gamma^{4}}\;$$

where h,g,f and m are arbitrary functions of *t*, the dots above the functions are the derivatives of the functions with respect to *t.* It is clear that the symmetries Eqs. 38-41 are just the special cases of Eqs. 46-49 for h=g=f=m=1.

## 5 Symmetries of integrable discrete systems

The discrete Kadomtsev-Petviashvili equation (dKP) equation was proposed independently by Hirota [30] and Miwa [31] in early 1980s so it is also called Hirota-Miwa (HM) equation. It is the most fundamental equation for discrete integrable system. We can use dKP equation as an example to explain the symmetry of discrete integrable systems. The dKP equation is a three-dimensional discrete integrable system with complex-valued *τ* -function defined on a three-dimensional lattice (*k*_1_*, k*_2_*, k*_3_) with lattice constants *a*_1_*, a*_2_*, a*_3_ such that *τ* (*k*_1_*, k*_2_*, k*_3_) = *τ* (*k*_1_*a*_1_*, k*_2_*a*_2_*, k*_3_*a*_3_). The dKP equation is one of the most fundamental equations in integrable system, which is usually expressed as a bilinear form:

*a*_1_(*a*_2_ *− a*_3_)*τ* (*k*_1_ + 1*, k*_2_*_,_k*_3_)*τ* (*k*_1_*, k*_2_ + 1*, k*_3_ + 1)
+*a*_2_(*a*_3_ *− a*_1_)*τ* (*k*_1_*, k*_2_ + 1*, k*_3_)*τ* (*k*_1_ + 1*, k*_2_*, k*_3_ + 1)
+*a*_3_(*a*_1_*−a*_2_)*τ*(*k*_1_*,k*_2_*,k*_3_+1)*τ*(*k*_1_+1*,k*_2_+1*,k*_3_) = 0 (50)

Here each subscript *i* denotes a forward shift in the corresponding discrete variable *n_i_*, for example, *τ_i_* = *τ* (*k_i_* + 1*, k_j_, k_m_*). Its geometric interpretation is given in [32].

If we use the following abbreviations:

*τ* (*k*_1_ + 1*, k*_2_*_,_k*_3_) = *τ*_1_*, τ* (*k*_1_*, k*_2_ + 1*, k*_3_ + 1) = *τ*_23_ then the dKP equation takes a short form:

(51) $$\;a_{1}\left(a_{2}-a_{3}\right)\tau_{1}\tau_{23}+a_{2}\left(a_{3}-a_{1}\right)\tau_{2}\tau_{31}+a_{3}\left(a_{1}-a_{2}\right)\tau_{3}\tau_{12}=0\;$$

Discrete KP hierarchy is an infinite number of bilinear equations with (*k_i_, k_j_, k_m_*) taken from (*k*_1_*, k*_2_*, k*_3_*, …*):

(52) $$\left(a_{j}^{-1}-a_{m}^{-1}\right)\tau_{i}\tau_{jm}+\left(a_{m}^{-1}-a_{i}^{-1}\right)\tau_{j}\tau_{mi}+\left(a_{i}^{-1}-a_{j}^{-1}\right)\tau_{m}\tau_{ij}=0$$

This hierarchy reflects the nature of symmetry of discrete integrable systems, which is called four-dimensional (4D) consistency or multi-dimensional consistency. From this symmetry, we can construct Darboux transformation, as well as conservation laws, of discrete integrable systems.

If we use the gauge transformation, the dKP equation takes a simpler form:

(53) $$\tau_{1}\tau_{23}-\tau_{2}\tau_{31}+\tau_{3}\tau_{12}=0\;$$

The Lax pair of the dKP equation was firstly proposed by Nimmo [33]. It can be derived from the dKP hierarchy:

(54) $$\;\tau_{m}\tau_{ij}-\tau_{j}\tau_{im}+\tau_{i}\tau_{jm}=0\;$$

Define:

$$\;\phi =\frac{\tau_{m}}{\tau },\;u^{ij}=\text{sgn}\left(j-i\right)\frac{\tau_{ij}\tau }{\tau_{i}\tau_{j}}$$

then it follows:

$$\phi_{i}-\phi_{j}=u^{ij}\phi$$

which is the Lax pair of the dKP hierarchy. By taking 1≤i,j≤3, we can have the following lattice:

$$u^{12}-u^{13}+u^{23}=0$$

From the compatibility condition, which is nothing but the dKP Eq. 53.

If we restore lattice constants, then we have the following linear problem:

$$\phi_{i}-\phi_{j}=\left(a_{i}^{-1}-a_{j}^{-1}\right)u^{ij}\phi$$

The bilinear equation of the discrete BKP was proposed by Miwa [31], [32], [33]:

(55) $$\begin{array}{ccc} & & \left(a_{2}-a_{3}\right)\left(a_{1}+a_{2}\right)\left(a_{1}+a_{3}\right)\tau_{1}\tau_{23}+\left(a_{3}-a_{1}\right)\left(a_{1}+a_{2}\right)\left(a_{2}+a_{3}\right)\tau_{2}\tau_{31}\\ & & +\;\left(a_{1}-a_{2}\right)\left(a_{3}+a_{2}\right)\left(a_{1}+a_{3}\right)\tau_{3}\tau_{12}\\ & & +\left(a_{2}-a_{3}\right)\left(a_{1}+a_{2}\right)\left(a_{1}+a_{3}\right)\tau \tau_{123}=0 \end{array}$$

which can be normalized into:

(56) $$\tau_{1}\tau_{23}+\tau_{2}\tau_{31}+\;\tau_{3}\tau_{12}+\tau \tau_{123}=0\;$$

Here we present the Lax pair of discrete BKP equation:

(57) $$\Phi_{12}-\Phi =\rho_{12}\left(\Phi_{1}-\Phi_{2}\right)\;$$

(58) $$\Phi_{23}-\Phi =\rho_{23}\left(\Phi_{2}-\Phi_{3}\right)\;$$

(59) $$\Phi_{31}-\Phi =\rho_{31}\left(\Phi_{3}-\Phi_{1}\right)\;$$

where $\rho_{31}=\tau_{i}\tau_{j}/\left(\tau_{ij}\tau \right).$ Shifting one more step in each equation, we have:

$$\Phi_{123}-\Phi_{3}=\rho_{123}\left(\Phi_{13}-\Phi_{23}\right)$$

$$\Phi_{123}-\Phi_{1}=\rho_{231}\left(\Phi_{12}-\Phi_{13}\right)$$

$$\Phi_{123}-\Phi_{2}=\rho_{312}\left(\Phi_{23}-\Phi_{12}\right)$$

Elimination of Φ123, we obtain:

$$\left(1+\rho_{123}\rho_{31}+\rho_{231}\rho_{31}+\rho_{123}\rho_{23}\right)\left(\Phi_{1}-\Phi_{3}\right)\;=1$$

which is actually the discrete BKP equation.

## 6 Conservation laws and deformations

It is known that a symmetry usually related to a conservation law. For instance, the space translation invariance is related to the moment conservation and the time translation is corresponding to the energy conservation. There are several traditional methods to find conservation laws from symmetries and the conservation laws can be applied to solve nonlinear physical problems. In this section, we apply the conservation laws to find higher dimensional integrable systems by using a deformation algorithm [34], [35], [36].

Deformation algorithm. For a general (1 + 1)-dimensional integrable local evolution system:

(60) $$u_{t}=F\left(u,\;u_{x},\;\ldots ,\;u_{x^{n}}\right),\;u_{x^{n}}=\partial_{x}^{n}u,\;u=\left(u_{1},\;u_{2},\ldots ,\;u_{m}\right)$$

if there exist some conservation laws:

(61) $$\rho_{it}=J_{ix},\;i=1,\;2,\;\ldots ,\;D-1,\;\rho_{i}=\rho_{i}\left(u),\;J_{i}=J_{i}(u,\;u_{x},\;\ldots ,\;u_{x^{N}}\right)\;$$

where the conserved densities $\rho_{i}$ are dependent only on the field u while the flows $J_{i}$ can be field derivative dependent, then the deformed (*D* + 1)-dimensional system:

(62) $$\hat{T}u=F\left(u,\;\hat{L}u,\;\ldots ,\;{\hat{L}}^{n}u\right)$$

is integrable with the deformation operators:

(63) $$\hat{L}\equiv \partial_{x}+\sum_{i=1}^{D-1}\rho_{i}\partial_{x_{i}},\;\hat{T}\equiv \partial_{t}+\sum_{i=1}^{D-1}{\bar{J}}_{i}\partial_{x_{i}}\;$$

and the deformed flows:

(64) $${\bar{J}}_{i}\equiv J_{i}{|}_{u_{x^{j}}\to {\hat{L}}^{j}u,\;j=1,\;2,\;\ldots ,N}$$

Applying the algorithm to known integrable systems such as the KdV equation [34], the nonlinear Schrödinger (NLS) equation [35] and the Camassa-Holm (CH) equation [36] etc., various higher dimensional integrable systems have been obtained.

For the KdV Eq. 1 with the conserved density *u*, we have the following (2 + 1)-dimensional integrable KdV-Harry-Dym (KdV-HD) equation [34]:

(65) $$u_{t}+{\left(u_{xx}-3u^{2}+3uu_{xy}+\frac{3}{2}u^{2}u_{yy}\right)}_{x}+{\left(u^{3}u_{yy}-u^{3}+\frac{3}{2}u^{2}u_{xy}\right)}_{y}=0$$

owing to the deformation algorithm. For the real shallow water waves, they are usually not center symmetric. The solitary waves of the (2 + 1)-dimensional model possesses asymmetric and the folded solitary wave structure because of the inclusion of nonlinear dispersion effects expressed by $u^{3}u_{yyy}$ which is more reasonable to describe the real water waves.

## 7 Ren symmetries and ren integrable systems

For a Lax integrable system, there are one or more Lax pairs. For instance, for the KdV Eq. 1, the related Lax pair possesses the form:

(66) $$L_{x}\psi \equiv \left(\partial_{x}^{2}-u+\lambda \right)\psi =0\;$$

(67) $$L_{t}\psi \equiv \left(\partial_{t}+4\partial_{x}^{3}-6u\partial_{x}-3u_{x}\right)\psi =0\;$$

where λ is an arbitrary spectral parameter and ψ is a spectral function related to λ. The KdV Eq. 1 is equivalent to the compatibility condition of Eq. 66 and Eq. 67:

$$\left[L_{x},\;L_{t}\right]\equiv L_{x}L_{t}-L_{t}L_{x}=0$$

If ψ is a bosonic function, then one can check that the square eigenfunction ${\left(\psi^{2}\right)}_{x}$ is a nonlocal symmetry of the KdV Eq. 1. Thus, one can construct some new integrable systems with help of the square eigenfunction symmetries, say, the second type of integrable KdV equation with self-consistent source:

(68) $$u_{t}+u_{xxx}-6uu_{x}={\left(\psi^{2}\right)}_{x}\;$$

(69) $$\left(\partial_{t}+4\partial_{x}^{3}-6u\partial_{x}-3u_{x}\right)\psi =0\;$$

If ψ=ξ of Eq. 66 and Eq. 67 is fermionic, then one can check that $\xi \xi_{xx}$ is a symmetry of the KdV equation. Therefore, the corresponding source equation is just the so-called super-integrable equation proposed by Kupershmidt [11]:

(70) $$u_{t}+u_{xxx}-6uu_{x}=\xi \xi_{xx}\;$$

(71) $$\left(\partial_{t}+4\partial_{x}^{3}-6u\partial_{x}-3u_{x}\right)\xi =0\;$$

Furthermore, the spectral function, ψ=ζ, of the Lax pair Eq. 66 and Eq. 67 may be a ren-number with the property:

(72) $$\begin{array}{c} \zeta^{\alpha }=0,\;\zeta^{j}\neq 0,\;j\;<\;\alpha \; \end{array}$$

where α is an arbitrary integer. “Ren” means arbitrary in Chinese.

When α=2, the ren-number defined by Eq. 72 is just the usual Grassmann number to describe fermions. The commutator between two ren-numbers θ and ζ possesses the form:

(73) $$\zeta \theta =q^{\beta }\theta \zeta ,\;q=\exp\left(\frac{2\pi i}{\alpha }\right),\;i\equiv \sqrt{-1},\;\beta =1,\;2,\;\ldots ,\;\alpha -1\;$$

β is called the degree of the ren-number of ζ, where we always fix the degree of θ as one later.

If $\psi =\zeta_{1}$ and $\psi =\zeta_{2}$ of the Lax pair Eq. 66 and Eq. 67 with the same spectral parameter λ, and the orders β and α−β, then one can check that $\zeta_{1}\zeta_{2xx}-\zeta_{1xx}\zeta_{2}$ is a symmetry of the KdV equation. Thus, we call the system of the following second types of source equation system:

(74) $$u_{t}+u_{xxx}-6uu_{x}+12\zeta_{1}\zeta_{2xx}-12\zeta_{1xx}\zeta_{2}=0\;$$

(75) $$\left(\partial_{t}+4\partial_{x}^{3}-6u\partial_{x}-3u_{x}\right)\zeta_{1}=0\;$$

(76) $$\left(\partial_{t}+4\partial_{x}^{3}-6u\partial_{x}-3u_{x}\right)\zeta_{2}=0\;$$

as the ren-integrable system. The Lax pair of (74)-(76) can be written as:

(77) $$\left(\partial_{x}^{2}-u+\lambda \right)\psi -\zeta_{1}\partial_{x}^{-1}\left(\zeta_{2}\psi \right)+\left(\partial_{x}^{-1}\zeta_{1}\psi \right)\;\zeta_{2}\;=0\;$$

(78) $$\left(\partial_{t}+4\partial_{x}^{3}-6u\partial_{x}-3u_{x}\right)\psi =0\;$$

To prove the Lax integrability of Eqs. 74-76, there is no any additional conditions except for the bosonic conditions of every term of Eq. 74 which requires the order of the $\zeta_{1}$ and $\zeta_{2}$ should be β and α−β respectively. When α=2, the ren-integrable system Eqs. 74-76 becomes a super-integrable system. From the commutation relation Eq. 73, we know that the ren integrable system can be used to describe the interactions among bosonic field u and the anyonic fields $\zeta_{1}$ and $\zeta_{2}$.

## 8 Supersymmetric and ren-symmetric integrable systems

In addition to the super-integrable systems, there are some types of super-symmetric integrable systems with the super-symmetric fields and supersymmetric derivative:

(79) $$D=\partial_{\theta }+\theta \partial_{x},\;D^{2}=\partial_{x}\;$$

The supersymmetric derivative operator D is invariant under the supersymmetric transformation:

(80) θ→θ+η,x→x−θη

After introducing the super-space variable θ and the supersymmetric derivative D, various usual bosonic integrable systems have been extended to the supersymmetric integrable systems. For instance, one of the supersymmetric N=1 KdV equation possesses the form (Φ≡Φ(x,t,θ)):

(81) $$\Phi_{t}+\Phi_{xxx}+a\left(D\Phi_{x}\right)\Phi +\left(6-a\right)\left(D\Phi \right)\Phi_{x}=0\;$$

Mathieu [30] had proven that the supersymmetric KdV equation is integrable only for a=0 and 3.

Similarly, for the ren functions, we may introduce ren-symmetric derivative:

(82) $$ℜ=\partial_{\theta }+\frac{\theta^{\alpha -1}}{{\left[\left(\alpha -1\right)!\right]}_{q}}\partial_{x},\;{ℜ}^{\alpha }=\partial_{x}\;$$

where ${\left[n!\right]}_{q}$ is defined as:

(83) $${\left[n!\right]}_{q}=\prod_{i=1}^{n}\frac{1-q^{i}}{1-q}=\prod_{i=1}^{n}i_{q}=1_{q}2_{q}\cdots n_{q},\;\;i_{q}=1+q+q^{2}+\cdots +q^{i-1}$$

It is not difficult to verify that the ren-symmetric derivative ℜ defined in Eq. 76 is invariant under the following ren-symmetric transformation:

(84) $$\theta \to \theta +\eta ,\;x\to x-f\left(\theta ,\;\eta \right),\;f=f\left(\theta ,\;\eta \right)=\sum_{k=1}^{\alpha -1}\frac{\theta^{k}\eta^{\alpha -k}}{{\left[k!\right]}_{q}{\left[\left(\alpha -k\right)!\right]}_{q}}\;$$

When α=2, the ren-symmetric derivative Eq. 82 and the ren-symmetric transformation Eq. 84 are reduced to the usual supersymmetric derivative Eq. 79 and the supersymmetric transformation Eq. 81, respectively.

Applying the ren-symmetric derivative Eq. 82 to the integrable systems, one can find various ren-symmetric integrable systems. For the KdV equation, the most general $\beta^{th}$ order ren-symmetric KdV equation may have the form, Φ=Φ(x,t,θ):

(85) $$\Phi_{t}+\Phi_{xxx}+\sum_{i=0}^{\left[\frac{\beta +\alpha }{2}\right]}a_{i}\left({ℜ}^{i}\Phi \right)\left({ℜ}^{\beta +\alpha -i}\Phi \right)=0,\;\beta =0,\;1,\;2,\ldots ,\;\alpha -1$$

where $a_{i},\;i=0,\;1,\;2,\;\ldots ,\;\left[\frac{\beta +\alpha }{2}\right]$, are bosonic constants, Φ is a $\beta^{th}$ order ren-field (ren-field with degree β) and $\left[\frac{\beta +\alpha }{2}\right]$ is the integer part of $\frac{\beta +\alpha }{2}.$

As in the super-symmetric (α=2) case, one may find some possible integrable cases by fixing the constants $a_{i}$ of the ren-symmetric KdV equation Eq. 85. For instance, for α=3, there are at least two ren-symmetric integrable KdV equation:

(86) $$\Phi_{1t}=-\Phi_{1xxx}+{\left({ℜ}^{2}\Phi_{1}\right)}^{2}+\left(ℜ\Phi_{1}\right)\Phi_{1x}$$

(87) $$\Phi_{2t}=-\Phi_{2xxx}+3\Phi_{2x}{ℜ}^{2}\Phi_{2}$$

where $\Phi_{1}\equiv \Phi_{1}\left(x,t,\theta \right)$ and $\Phi_{2}\equiv \Phi_{2}\left(x,t,\theta \right)$ are the first and second order ren fields for α=3 with the properties:$\;\theta^{3}=\Phi_{1}^{3}=\Phi_{2}^{3}=0,\;\Phi_{1}\theta =q\theta \Phi_{1},\;\Phi_{2}\theta =q^{2}\theta \Phi_{2},\;q=\exp\left(\frac{2\pi i}{3}\right),\;\;ℜ=\partial_{\theta }-q\theta^{2}\partial_{x},\;\text{and}\;{ℜ}^{3}=\partial_{x}$.

## 9 Summary and discussions

In summary, symmetries are very important in natural science especially in integrable systems because of the existence of infinitely many symmetries. Usually, to find exact solutions related to symmetries, the symmetries are restricted to the Lie point symmetries. Applying Lie point symmetries to nonlinear systems, one may find more general solutions from a special one. One can also find group invariant solutions with related symmetry reductions. In addition to the Lie point symmetries, there are many generalized local and nonlocal symmetries which can be used to find algebro-geometric solutions. The symmetries are related to conservation laws because of the Noether's theorem. Symmetries can also be used to find new integrable systems by means of the nonlinearization approach, introducing self-consistent sources, symmetry constraints and reductions. To find infinitely many symmetries there are some different approaches including the recursion operators, the master-symmetry approach, the series symmetry method, the residual symmetry method, the duality method [21] and so on. For the discrete integrable systems, we discuss the symmetry in an alternative way by considering the hierarchy in bilinear forms because the hierarchy reflects the nature of symmetry of discrete integrable systems.

Conservation laws can also be used to study nonlinear physics in some different ways. In this paper, a novel idea on the conservation laws is proposed to find higher dimensional integrable systems from lower dimensional ones by means of a deformation algorithm [34], [35], [36].

After introducing anti-commuted Grassmann variables, the usual bosonic systems may have additional symmetries related to the Grassmannian spectral functions, which lead to the appearance of the super-integrable systems. Additionally, one can introduce the symmetry between bosons and fermions, which yields the various supersymmetric integrable systems [37], [38], [39], [40], [41]. Furthermore, by introducing non-commute ren variables [42], we have found some ren integrable and ren-symmetric integrable models which may be used to describe anyon physics and provide some more candidates on dark matters.

The ren-variables or anyonic variables introduced in this paper and Ref. [42] are subject to braid statistics of fractional charges and hence are of the general type encountered in some aspects of anyonic physics. A similar algebra named braided algebra or anyonic algebra has been introduced in Ref. [43] as a braided-Hopf algebra [44]. In order to study the integrable systems related to the anyon physics, we should point out that our ren-algebra is different from that of [43] because of the introduction of degrees of the anyonic variables. Our commutation relation (72) is degree-dependent while the commutation relations of Ref. [43] is only same as ours for degree 1. Furthermore, we have introduced the ren-symmetric derivative ℜ (see Eqs. 82-84) such that the supersymmetric integrable systems can be extended to ren-symmetric integrable systems. Though we have successfully introduced some ren-integrable systems and ren-symmetric integrable systems, it is still open to derive these types of integrable models from real physical systems even if for special fermionic case because the Grassmann number and the ren-numbers are only the classical aspect while the fermions and the anyons are essential valid only in quantum physics.

## CRediT authorship contribution statement

**Sen-Yue Lou:** Conceptualization, Formal analysis, Investigation, Methodology, Project administration, Writing – original draft, Writing – review & editing. **Bao-Feng Feng:** Conceptualization, Formal analysis, Investigation, Methodology, Project administration, Writing – original draft, Writing – review & editing.
